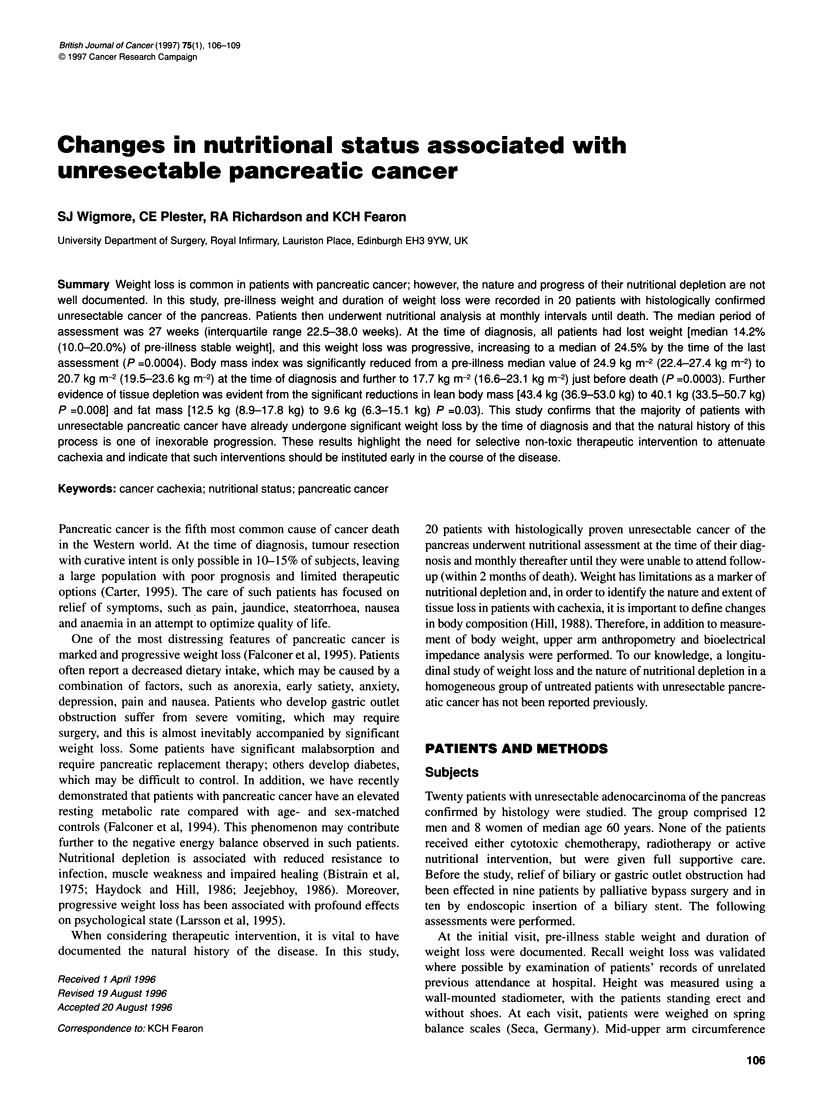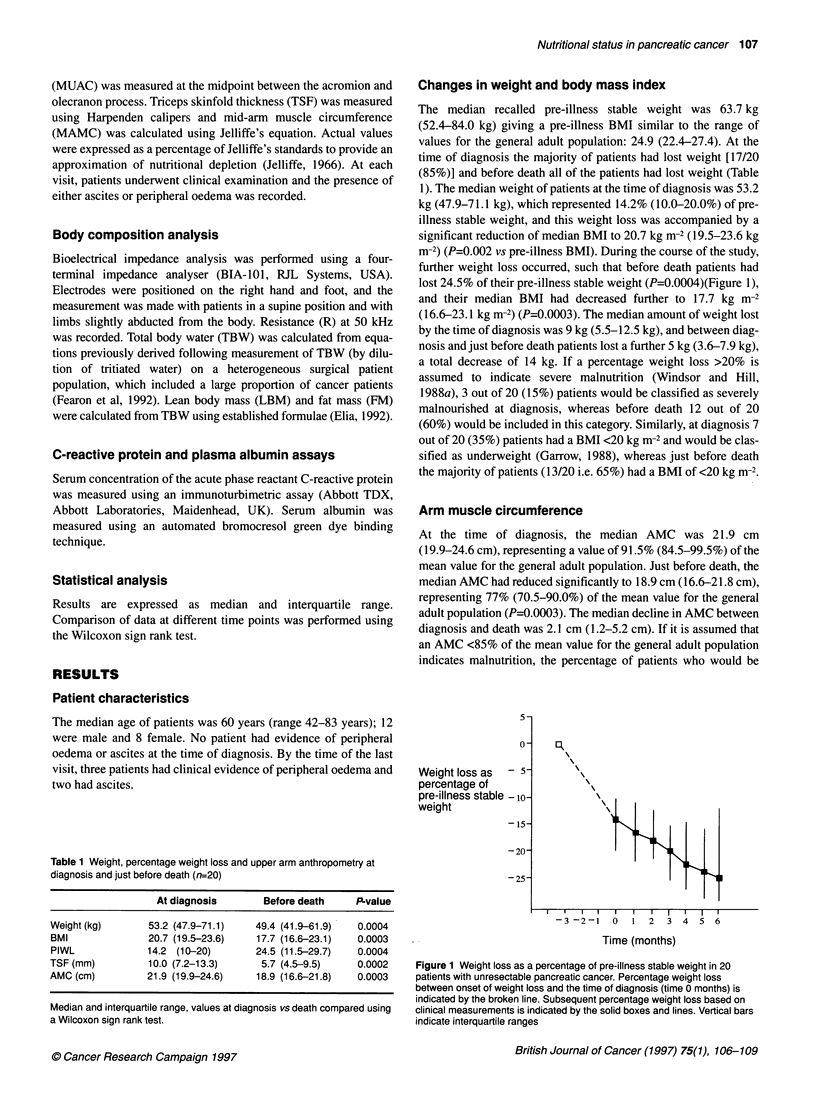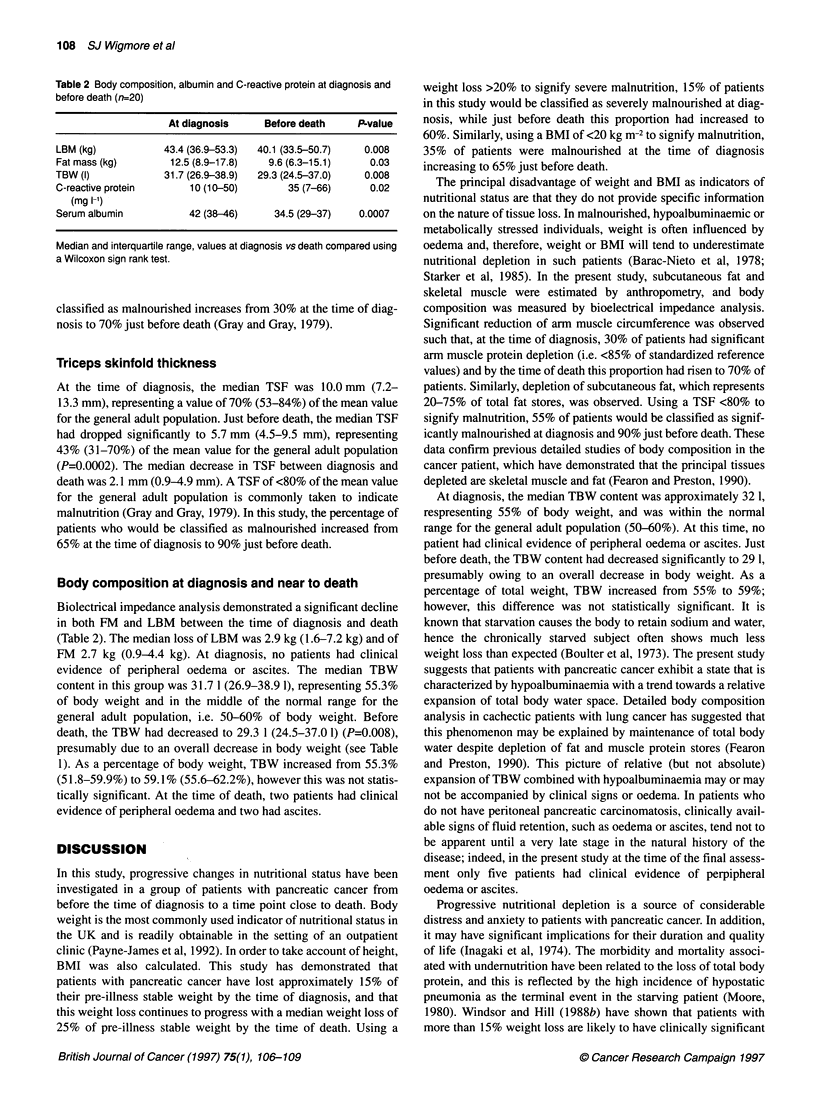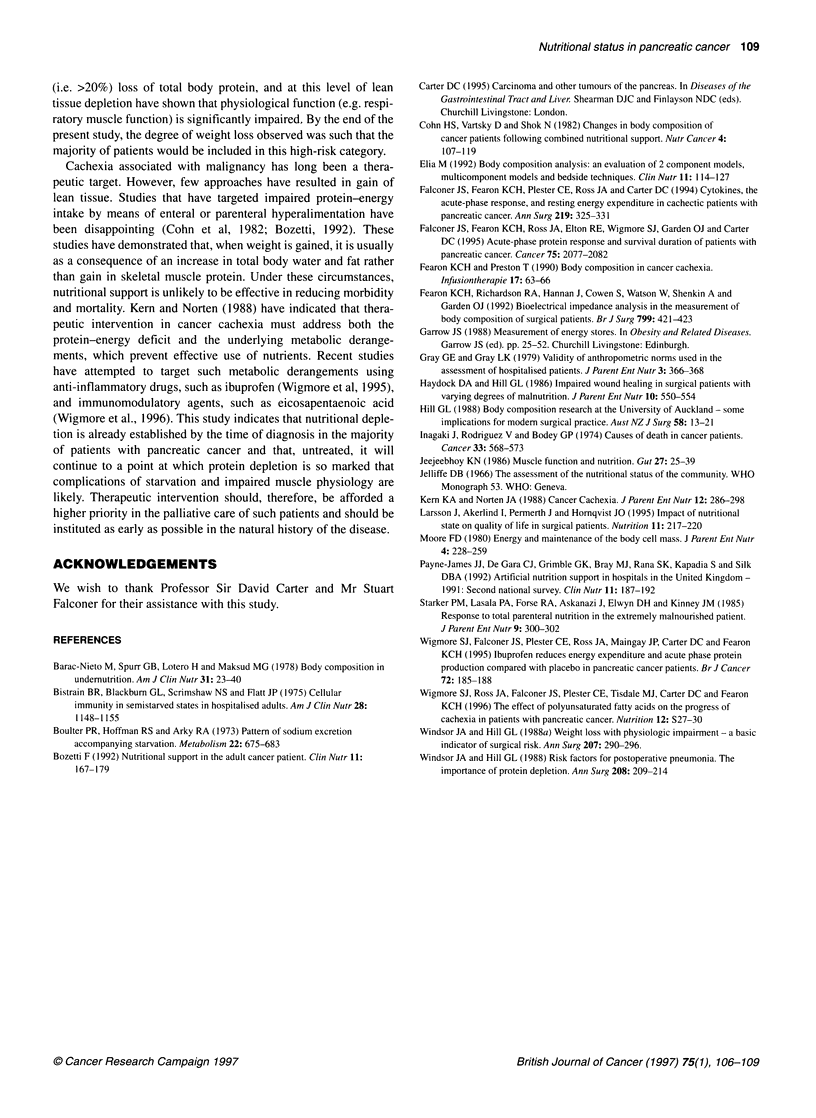# Changes in nutritional status associated with unresectable pancreatic cancer.

**DOI:** 10.1038/bjc.1997.17

**Published:** 1997

**Authors:** S. J. Wigmore, C. E. Plester, R. A. Richardson, K. C. Fearon

**Affiliations:** University Department of Surgery, Royal Infirmary, Edinburgh, UK.

## Abstract

Weight loss is common in patients with pancreatic cancer; however, the nature and progress of their nutritional depletion are not well documented. In this study, pre-illness weight and duration of weight loss were recorded in 20 patients with histologically confirmed unresectable cancer of the pancreas. Patients then underwent nutritional analysis at monthly intervals until death. The median period of assessment was 27 weeks (interquartile range 22.5-38.0 weeks). At the time of diagnosis, all patients had lost weight [median 14.2% (10.0-20.0%) of pre-illness stable weight], and this weight loss was progressive, increasing to a median of 24.5% by the time of the last assessment (P =0.0004). Body mass index was significantly reduced from a pre-illness median value of 24.9 kg m-2 (22.4-27.4 kg m-2) to 20.7 kg m-2 (19.5-23.6 kg m-2) at the time of diagnosis and further to 17.7 kg m-2 (16.6-23.1 kg m-2) just before death (P =0.0003). Further evidence of tissue depletion was evident from the significant reductions in lean body mass [43.4 kg (36.9-53.0 kg) to 40.1 kg (33.5-50.7 kg) P =0.008] and fat mass [12.5 kg (8.9-17.8 kg) to 9.6 kg (6.3-15.1 kg) P =0.03). This study confirms that the majority of patients with unresectable pancreatic cancer have already undergone significant weight loss by the time of diagnosis and that the natural history of this process is one of inexorable progression. These results highlight the need for selective non-toxic therapeutic intervention to attenuate cachexia and indicate that such interventions should be instituted early in the course of the disease.


British Journal of Cancer (1997) 75(1), 106–109
© 1997 Cancer Research Campaign

### Changes in nutritional status associated with unresectable pancreatic cancer

**SJ Wigmore, CE Plester, RA Richardson and KCH Fearon**

University Department of Surgery, Royal Infirmary, Lauriston Place, Edinburgh EH3 9YW, UK

**Summary** Weight loss is common in patients with pancreatic cancer; however, the nature and progress of their nutritional depletion are not well documented. In this study, pre-illness weight and duration of weight loss were recorded in 20 patients with histologically confirmed unresectable cancer of the pancreas. Patients then underwent nutritional analysis at monthly intervals until death. The median period of assessment was 27 weeks (interquartile range 22.5–38.0 weeks). At the time of diagnosis, all patients had lost weight [median 14.2% (10.0–20.0%) of pre-illness stable weight], and this weight loss was progressive, increasing to a median of 24.5% by the time of the last assessment ($P$ =0.0004). Body mass index was significantly reduced from a pre-illness median value of 24.9 kg m$^{-2}$ (22.4–27.4 kg m$^{-2}$) to 20.7 kg m$^{-2}$ (19.5–23.6 kg m$^{-2}$) at the time of diagnosis and further to 17.7 kg m$^{-2}$ (16.6–23.1 kg m$^{-2}$) just before death ($P$ =0.0003). Further evidence of tissue depletion was evident from the significant reductions in lean body mass [43.4 kg (36.9–53.0 kg) to 40.1 kg (33.5–50.7 kg) $P$ =0.008] and fat mass [12.5 kg (8.9–17.8 kg) to 9.6 kg (6.3–15.1 kg) $P$ =0.03). This study confirms that the majority of patients with unresectable pancreatic cancer have already undergone significant weight loss by the time of diagnosis and that the natural history of this process is one of inexorable progression. These results highlight the need for selective non-toxic therapeutic intervention to attenuate cachexia and indicate that such interventions should be instituted early in the course of the disease.

**Keywords:** cancer cachexia; nutritional status; pancreatic cancer

Pancreatic cancer is the fifth most common cause of cancer death in the Western world. At the time of diagnosis, tumour resection with curative intent is only possible in 10–15% of subjects, leaving a large population with poor prognosis and limited therapeutic options (Carter, 1995). The care of such patients has focused on relief of symptoms, such as pain, jaundice, steatorrhoea, nausea and anaemia in an attempt to optimize quality of life.

One of the most distressing features of pancreatic cancer is marked and progressive weight loss (Falconer et al, 1995). Patients often report a decreased dietary intake, which may be caused by a combination of factors, such as anorexia, early satiety, anxiety, depression, pain and nausea. Patients who develop gastric outlet obstruction suffer from severe vomiting, which may require surgery, and this is almost inevitably accompanied by significant weight loss. Some patients have significant malabsorption and require pancreatic replacement therapy; others develop diabetes, which may be difficult to control. In addition, we have recently demonstrated that patients with pancreatic cancer have an elevated resting metabolic rate compared with age- and sex-matched controls (Falconer et al, 1994). This phenomenon may contribute further to the negative energy balance observed in such patients. Nutritional depletion is associated with reduced resistance to infection, muscle weakness and impaired healing (Bistrain et al, 1975; Haydock and Hill, 1986; Jeejebhoy, 1986). Moreover, progressive weight loss has been associated with profound effects on psychological state (Larsson et al, 1995).

When considering therapeutic intervention, it is vital to have documented the natural history of the disease. In this study, 20 patients with histologically proven unresectable cancer of the pancreas underwent nutritional assessment at the time of their diagnosis and monthly thereafter until they were unable to attend follow-up (within 2 months of death). Weight has limitations as a marker of nutritional depletion and, in order to identify the nature and extent of tissue loss in patients with cachexia, it is important to define changes in body composition (Hill, 1988). Therefore, in addition to measurement of body weight, upper arm anthropometry and bioelectrical impedance analysis were performed. To our knowledge, a longitudinal study of weight loss and the nature of nutritional depletion in a homogeneous group of untreated patients with unresectable pancreatic cancer has not been reported previously.

*Received 1 April 1996*
*Revised 19 August 1996*
*Accepted 20 August 1996*

*Correspondence to:* KCH Fearon

### PATIENTS AND METHODS

### Subjects

Twenty patients with unresectable adenocarcinoma of the pancreas confirmed by histology were studied. The group comprised 12 men and 8 women of median age 60 years. None of the patients received either cytotoxic chemotherapy, radiotherapy or active nutritional intervention, but were given full supportive care. Before the study, relief of biliary or gastric outlet obstruction had been effected in nine patients by palliative bypass surgery and in ten by endoscopic insertion of a biliary stent. The following assessments were performed.

At the initial visit, pre-illness stable weight and duration of weight loss were documented. Recall weight loss was validated where possible by examination of patients' records of unrelated previous attendance at hospital. Height was measured using a wall-mounted stadiometer, with the patients standing erect and without shoes. At each visit, patients were weighed on spring balance scales (Seca, Germany). Mid-upper arm circumference

(MUAC) was measured at the midpoint between the acromion and olecranon process. Triceps skinfold thickness (TSF) was measured using Harpenden calipers and mid-arm muscle circumference (MAMC) was calculated using Jelliffe's equation. Actual values were expressed as a percentage of Jelliffe's standards to provide an approximation of nutritional depletion (Jelliffe, 1966). At each visit, patients underwent clinical examination and the presence of either ascites or peripheral oedema was recorded.

### Body composition analysis

Bioelectrical impedance analysis was performed using a four-terminal impedance analyser (BIA-101, RJL Systems, USA). Electrodes were positioned on the right hand and foot, and the measurement was made with patients in a supine position and with limbs slightly abducted from the body. Resistance (R) at 50 kHz was recorded. Total body water (TBW) was calculated from equations previously derived following measurement of TBW (by dilution of tritiated water) on a heterogeneous surgical patient population, which included a large proportion of cancer patients (Fearon et al, 1992). Lean body mass (LBM) and fat mass (FM) were calculated from TBW using established formulae (Elia, 1992).

### C-reactive protein and plasma albumin assays

Serum concentration of the acute phase reactant C-reactive protein was measured using an immunoturbimetric assay (Abbott TDX, Abbott Laboratories, Maidenhead, UK). Serum albumin was measured using an automated bromocresol green dye binding technique.

### Statistical analysis

Results are expressed as median and interquartile range. Comparison of data at different time points was performed using the Wilcoxon sign rank test.

### RESULTS

### Patient characteristics

The median age of patients was 60 years (range 42–83 years); 12 were male and 8 female. No patient had evidence of peripheral oedema or ascites at the time of diagnosis. By the time of the last visit, three patients had clinical evidence of peripheral oedema and two had ascites.

**Table 1** Weight, percentage weight loss and upper arm anthropometry at diagnosis and just before death (*n*=20)

| | At diagnosis | Before death | *P*-value |
|---|---|---|---|
| Weight (kg) | 53.2 (47.9–71.1) | 49.4 (41.9–61.9) | 0.0004 |
| BMI | 20.7 (19.5–23.6) | 17.7 (16.6–23.1) | 0.0003 |
| PIWL | 14.2 (10–20) | 24.5 (11.5–29.7) | 0.0004 |
| TSF (mm) | 10.0 (7.2–13.3) | 5.7 (4.5–9.5) | 0.0002 |
| AMC (cm) | 21.9 (19.9–24.6) | 18.9 (16.6–21.8) | 0.0003 |

Median and interquartile range, values at diagnosis *vs* death compared using a Wilcoxon sign rank test.

### Changes in weight and body mass index

The median recalled pre-illness stable weight was 63.7 kg (52.4–84.0 kg) giving a pre-illness BMI similar to the range of values for the general adult population: 24.9 (22.4–27.4). At the time of diagnosis the majority of patients had lost weight [17/20 (85%)] and before death all of the patients had lost weight (Table 1). The median weight of patients at the time of diagnosis was 53.2 kg (47.9–71.1 kg), which represented 14.2% (10.0–20.0%) of pre-illness stable weight, and this weight loss was accompanied by a significant reduction of median BMI to 20.7 kg $m^{-2}$ (19.5–23.6 kg $m^{-2}$) ($P$=0.002 *vs* pre-illness BMI). During the course of the study, further weight loss occurred, such that before death patients had lost 24.5% of their pre-illness stable weight ($P$=0.0004)(Figure 1), and their median BMI had decreased further to 17.7 kg $m^{-2}$ (16.6–23.1 kg $m^{-2}$) ($P$=0.0003). The median amount of weight lost by the time of diagnosis was 9 kg (5.5–12.5 kg), and between diagnosis and just before death patients lost a further 5 kg (3.6–7.9 kg), a total decrease of 14 kg. If a percentage weight loss >20% is assumed to indicate severe malnutrition (Windsor and Hill, 1988*a*), 3 out of 20 (15%) patients would be classified as severely malnourished at diagnosis, whereas before death 12 out of 20 (60%) would be included in this category. Similarly, at diagnosis 7 out of 20 (35%) patients had a BMI <20 kg $m^{-2}$ and would be classified as underweight (Garrow, 1988), whereas just before death the majority of patients (13/20 i.e. 65%) had a BMI of <20 kg $m^{-2}$.

### Arm muscle circumference

At the time of diagnosis, the median AMC was 21.9 cm (19.9–24.6 cm), representing a value of 91.5% (84.5–99.5%) of the mean value for the general adult population. Just before death, the median AMC had reduced significantly to 18.9 cm (16.6–21.8 cm), representing 77% (70.5–90.0%) of the mean value for the general adult population ($P$=0.0003). The median decline in AMC between diagnosis and death was 2.1 cm (1.2–5.2 cm). If it is assumed that an AMC <85% of the mean value for the general adult population indicates malnutrition, the percentage of patients who would be

**Figure 1** Weight loss as a percentage of pre-illness stable weight in 20 patients with unresectable pancreatic cancer. Percentage weight loss between onset of weight loss and the time of diagnosis (time 0 months) is indicated by the broken line. Subsequent percentage weight loss based on clinical measurements is indicated by the solid boxes and lines. Vertical bars indicate interquartile ranges

© Cancer Research Campaign 1997

**Table 2** Body composition, albumin and C-reactive protein at diagnosis and before death ($n$=20)

| | At diagnosis | Before death | P-value |
|---|---|---|---|
| LBM (kg) | 43.4 (36.9–53.3) | 40.1 (33.5–50.7) | 0.008 |
| Fat mass (kg) | 12.5 (8.9–17.8) | 9.6 (6.3–15.1) | 0.03 |
| TBW (l) | 31.7 (26.9–38.9) | 29.3 (24.5–37.0) | 0.008 |
| C-reactive protein (mg $l^{-1}$) | 10 (10–50) | 35 (7–66) | 0.02 |
| Serum albumin | 42 (38–46) | 34.5 (29–37) | 0.0007 |

Median and interquartile range, values at diagnosis *vs* death compared using a Wilcoxon sign rank test.

classified as malnourished increases from 30% at the time of diagnosis to 70% just before death (Gray and Gray, 1979).

### Triceps skinfold thickness

At the time of diagnosis, the median TSF was 10.0 mm (7.2–13.3 mm), representing a value of 70% (53–84%) of the mean value for the general adult population. Just before death, the median TSF had dropped significantly to 5.7 mm (4.5–9.5 mm), representing 43% (31–70%) of the mean value for the general adult population ($P$=0.0002). The median decrease in TSF between diagnosis and death was 2.1 mm (0.9–4.9 mm). A TSF of <80% of the mean value for the general adult population is commonly taken to indicate malnutrition (Gray and Gray, 1979). In this study, the percentage of patients who would be classified as malnourished increased from 65% at the time of diagnosis to 90% just before death.

### Body composition at diagnosis and near to death

Biolectrical impedance analysis demonstrated a significant decline in both FM and LBM between the time of diagnosis and death (Table 2). The median loss of LBM was 2.9 kg (1.6–7.2 kg) and of FM 2.7 kg (0.9–4.4 kg). At diagnosis, no patients had clinical evidence of peripheral oedema or ascites. The median TBW content in this group was 31.7 l (26.9–38.9 l), representing 55.3% of body weight and in the middle of the normal range for the general adult population, i.e. 50–60% of body weight. Before death, the TBW had decreased to 29.3 l (24.5–37.0 l) ($P$=0.008), presumably due to an overall decrease in body weight (see Table 1). As a percentage of body weight, TBW increased from 55.3% (51.8–59.9%) to 59.1% (55.6–62.2%), however this was not statistically significant. At the time of death, two patients had clinical evidence of peripheral oedema and two had ascites.

### DISCUSSION

In this study, progressive changes in nutritional status have been investigated in a group of patients with pancreatic cancer from before the time of diagnosis to a time point close to death. Body weight is the most commonly used indicator of nutritional status in the UK and is readily obtainable in the setting of an outpatient clinic (Payne-James et al, 1992). In order to take account of height, BMI was also calculated. This study has demonstrated that patients with pancreatic cancer have lost approximately 15% of their pre-illness stable weight by the time of diagnosis, and that this weight loss continues to progress with a median weight loss of 25% of pre-illness stable weight by the time of death. Using a weight loss >20% to signify severe malnutrition, 15% of patients in this study would be classified as severely malnourished at diagnosis, while just before death this proportion had increased to 60%. Similarly, using a BMI of <20 kg $m^{-2}$ to signify malnutrition, 35% of patients were malnourished at the time of diagnosis increasing to 65% just before death.

The principal disadvantage of weight and BMI as indicators of nutritional status are that they do not provide specific information on the nature of tissue loss. In malnourished, hypoalbuminaemic or metabolically stressed individuals, weight is often influenced by oedema and, therefore, weight or BMI will tend to underestimate nutritional depletion in such patients (Barac-Nieto et al, 1978; Starker et al, 1985). In the present study, subcutaneous fat and skeletal muscle were estimated by anthropometry, and body composition was measured by bioelectrical impedance analysis. Significant reduction of arm muscle circumference was observed such that, at the time of diagnosis, 30% of patients had significant arm muscle protein depletion (i.e. <85% of standardized reference values) and by the time of death this proportion had risen to 70% of patients. Similarly, depletion of subcutaneous fat, which represents 20–75% of total fat stores, was observed. Using a TSF <80% to signify malnutrition, 55% of patients would be classified as significantly malnourished at diagnosis and 90% just before death. These data confirm previous detailed studies of body composition in the cancer patient, which have demonstrated that the principal tissues depleted are skeletal muscle and fat (Fearon and Preston, 1990).

At diagnosis, the median TBW content was approximately 32 l, respesenting 55% of body weight, and was within the normal range for the general adult population (50–60%). At this time, no patient had clinical evidence of peripheral oedema or ascites. Just before death, the TBW content had decreased significantly to 29 l, presumably owing to an overall decrease in body weight. As a percentage of total weight, TBW increased from 55% to 59%; however, this difference was not statistically significant. It is known that starvation causes the body to retain sodium and water, hence the chronically starved subject often shows much less weight loss than expected (Boulter et al, 1973). The present study suggests that patients with pancreatic cancer exhibit a state that is characterized by hypoalbuminaemia with a trend towards a relative expansion of total body water space. Detailed body composition analysis in cachectic patients with lung cancer has suggested that this phenomenon may be explained by maintenance of total body water despite depletion of fat and muscle protein stores (Fearon and Preston, 1990). This picture of relative (but not absolute) expansion of TBW combined with hypoalbuminaemia may or may not be accompanied by clinical signs or oedema. In patients who do not have peritoneal pancreatic carcinomatosis, clinically available signs of fluid retention, such as oedema or ascites, tend not to be apparent until a very late stage in the natural history of the disease; indeed, in the present study at the time of the final assessment only five patients had clinical evidence of perpipheral oedema or ascites.

Progressive nutritional depletion is a source of considerable distress and anxiety to patients with pancreatic cancer. In addition, it may have significant implications for their duration and quality of life (Inagaki et al, 1974). The morbidity and mortality associated with undernutrition have been related to the loss of total body protein, and this is reflected by the high incidence of hypostatic pneumonia as the terminal event in the starving patient (Moore, 1980). Windsor and Hill (1988*b*) have shown that patients with more than 15% weight loss are likely to have clinically significant

© Cancer Research Campaign 1997

(i.e. >20%) loss of total body protein, and at this level of lean tissue depletion have shown that physiological function (e.g. respiratory muscle function) is significantly impaired. By the end of the present study, the degree of weight loss observed was such that the majority of patients would be included in this high-risk category.

Cachexia associated with malignancy has long been a therapeutic target. However, few approaches have resulted in gain of lean tissue. Studies that have targeted impaired protein–energy intake by means of enteral or parenteral hyperalimentation have been disappointing (Cohn et al, 1982; Bozetti, 1992). These studies have demonstrated that, when weight is gained, it is usually as a consequence of an increase in total body water and fat rather than gain in skeletal muscle protein. Under these circumstances, nutritional support is unlikely to be effective in reducing morbidity and mortality. Kern and Norten (1988) have indicated that therapeutic intervention in cancer cachexia must address both the protein–energy deficit and the underlying metabolic derangements, which prevent effective use of nutrients. Recent studies have attempted to target such metabolic derangements using anti-inflammatory drugs, such as ibuprofen (Wigmore et al, 1995), and immunomodulatory agents, such as eicosapentaenoic acid (Wigmore et al., 1996). This study indicates that nutritional depletion is already established by the time of diagnosis in the majority of patients with pancreatic cancer and that, untreated, it will continue to a point at which protein depletion is so marked that complications of starvation and impaired muscle physiology are likely. Therapeutic intervention should, therefore, be afforded a higher priority in the palliative care of such patients and should be instituted as early as possible in the natural history of the disease.

### ACKNOWLEDGEMENTS

We wish to thank Professor Sir David Carter and Mr Stuart Falconer for their assistance with this study.

### REFERENCES

Barac-Nieto M, Spurr GB, Lotero H and Maksud MG (1978) Body composition in undernutrition. *Am J Clin Nutr* **31:** 23–40

Bistrain BR, Blackburn GL, Scrimshaw NS and Flatt JP (1975) Cellular immunity in semistarved states in hospitalised adults. *Am J Clin Nutr* **28:** 1148–1155

Boulter PR, Hoffman RS and Arky RA (1973) Pattern of sodium excretion accompanying starvation. *Metabolism* **22:** 675–683

Bozetti F (1992) Nutritional support in the adult cancer patient. *Clin Nutr* **11:** 167–179

Carter DC (1995) Carcinoma and other tumours of the pancreas. In *Diseases of the Gastrointestinal Tract and Liver.* Shearman DJC and Finlayson NDC (eds). Churchill Livingstone: London.

Cohn HS, Vartsky D and Shok N (1982) Changes in body composition of cancer patients following combined nutritional support. *Nutr Cancer* **4:** 107–119

Elia M (1992) Body composition analysis: an evaluation of 2 component models, multicomponent models and bedside techniques. *Clin Nutr* **11:** 114–127

Falconer JS, Fearon KCH, Plester CE, Ross JA and Carter DC (1994) Cytokines, the acute-phase response, and resting energy expenditure in cachectic patients with pancreatic cancer. *Ann Surg* **219:** 325–331

Falconer JS, Fearon KCH, Ross JA, Elton RE, Wigmore SJ, Garden OJ and Carter DC (1995) Acute-phase protein response and survival duration of patients with pancreatic cancer. *Cancer* **75:** 2077–2082

Fearon KCH and Preston T (1990) Body composition in cancer cachexia. *Infusiontherapie* **17:** 63–66

Fearon KCH, Richardson RA, Hannan J, Cowen S, Watson W, Shenkin A and Garden OJ (1992) Bioelectrical impedance analysis in the measurement of body composition of surgical patients. *Br J Surg* **799:** 421–423

Garrow JS (1988) Measurement of energy stores. In *Obesity and Related Diseases.* Garrow JS (ed). pp. 25–52. Churchill Livingstone: Edinburgh.

Gray GE and Gray LK (1979) Validity of anthropometric norms used in the assessment of hospitalised patients. *J Parent Ent Nutr* **3:** 366–368

Haydock DA and Hill GL (1986) Impaired wound healing in surgical patients with varying degrees of malnutrition. *J Parent Ent Nutr* **10:** 550–554

Hill GL (1988) Body composition research at the University of Auckland – some implications for modern surgical practice. *Aust NZ J Surg* **58:** 13–21

Inagaki J, Rodriguez V and Bodey GP (1974) Causes of death in cancer patients. *Cancer* **33:** 568–573

Jeejeebhoy KN (1986) Muscle function and nutrition. *Gut* **27:** 25–39

Jelliffe DB (1966) The assessment of the nutritional status of the community. WHO Monograph 53. WHO: Geneva.

Kern KA and Norten JA (1988) Cancer Cachexia. *J Parent Ent Nutr* **12:** 286–298

Larsson J, Akerlind I, Permerth J and Hornqvist JO (1995) Impact of nutritional state on quality of life in surgical patients. *Nutrition* **11:** 217–220

Moore FD (1980) Energy and maintenance of the body cell mass. J *Parent Ent Nutr* **4:** 228–259

Payne-James JJ, De Gara CJ, Grimble GK, Bray MJ, Rana SK, Kapadia S and Silk DBA (1992) Artificial nutrition support in hospitals in the United Kingdom – 1991: Second national survey. *Clin Nutr* **11:** 187–192

Starker PM, Lasala PA, Forse RA, Askanazi J, Elwyn DH and Kinney JM (1985) Response to total parenteral nutrition in the extremely malnourished patient. *J Parent Ent Nutr* **9:** 300–302

Wigmore SJ, Falconer JS, Plester CE, Ross JA, Maingay JP, Carter DC and Fearon KCH (1995) Ibuprofen reduces energy expenditure and acute phase protein production compared with placebo in pancreatic cancer patients. *Br J Cancer* **72:** 185–188

Wigmore SJ, Ross JA, Falconer JS, Plester CE, Tisdale MJ, Carter DC and Fearon KCH (1996) The effect of polyunsaturated fatty acids on the progress of cachexia in patients with pancreatic cancer. *Nutrition* **12:** S27–30

Windsor JA and Hill GL (1988*a*) Weight loss with physiologic impairment – a basic indicator of surgical risk. *Ann Surg* **207:** 290–296.

Windsor JA and Hill GL (1988) Risk factors for postoperative pneumonia. The importance of protein depletion. *Ann Surg* **208:** 209–214

© Cancer Research Campaign 1997